# Relationship between workplace violence and depressive symptoms among emergency department physicians in China: the mediating role of occupational burnout

**DOI:** 10.7189/jogh.15.04259

**Published:** 2025-09-19

**Authors:** Ruofan Zhang, Ge Qu, Jing Feng, Yuchao Sun, Xinyan Li, Aoqi Shen, Heng Jiang, Yingbin Luo, Zixin Xu, Xingyue Song, Chuanzhu Lv, Yanli Zuo, Zhong Li, Yong Gan, Zuxun Lu

**Affiliations:** 1 Huazhong University of Science and Technology, Tongji Medical College, School of Public Health, Department of Social Medicine and Health Management, Wuhan, China; 2 La Trobe University, School of Psychology and Public Health, Department of Public Health, Melbourne, Australia; 3 University of Melbourne, Melbourne School of Population and Global Health, Melbourne, Australia; 4 Emergency Department of The Second Affiliated Hospital of Hainan Medical University, Key Laboratory of Emergency and Trauma of Ministry of Education, Haikou, China; 5 University of Electronic Science and Technology of China, Sichuan Provincial People's Hospital, Emergency Medicine Center, Chengdu, China; 6 Guangxi Medical University, School of General Practice, Nanning, China; 7 Nanjing Medical University, School of Health Policy and Management, Nanjing, China

## Abstract

**Background:**

Workplace violence (WPV) was a global public health issue worldwide. Given the specificity of emergency department physicians' professional roles, the incidence of WPV was significantly higher than that of practitioners in other healthcare groups. Investigating the association between exposure to WPV and psychological health can provide a scientific basis for managers to implement precise interventions on violent incidents, thereby effectively reducing the adverse impacts of WPV.

**Methods:**

A multi-stage random sampling method was employed to conduct a questionnaire survey among 14 848 emergency department physicians across 31 provincial-level administrative regions in China from July to September 2019. This survey utilised the WPV Scale, the Maslach Burnout Inventory-Human Services Survey (MBI-HSS), and the Center for Epidemiological Studies Depression Scale (CES-D) to investigate the levels of WPV, burnout, and depressive symptoms among respondents. Data analysis were performed using STATA 17.0 and Amos 26.0 software.

**Results:**

A total of 90.40% of emergency department physicians reported experiencing at least one incident of WPV in the past year. The mean score for depressive symptoms was 16.80 ± 14.78, and the mean score for burnout was 82.94 ± 26.11. The correlation coefficients between WPV and depressive symptoms, WPV and job burnout, and depressive symptoms and job burnout were 0.444, 0.347, and 0.562, respectively (all *P* < 0.01). Structural equation analysis indicated that the total effect of WPV on depressive symptoms was 0.471, with a mediating effect of burnout at 0.421, accounting for 89.38% of the total effect.

**Conclusions:**

Emergency department physicians had a severe level of depressive symptoms and burnout, with burnout playing a mediating role between WPV and depressive symptoms. Therefore, future policy efforts are warranted to build a supportive work environment for emergency department physicians and consequently enhance their well-being.

Workplace violence (WPV) in medical settings is a significant public health issue worldwide. According to the definition jointly released by World Health Organization (WHO), International Labour Office (ILO), International Council of Nurses (ICN) and Public Services International (PSI) in 2002, WPV was defined as ‘Incidents where staff are abused, threatened or assaulted in circumstances related to their work, including commuting to and from work, involving an explicit or implicit challenge to their safety, well-being or health (adapted from European Commission)’ [1]. Currently, WPV is one of the main challenges faced by health-care professionals globally. A meta-analysis by Liu et al. [2] showed that 61.9% (95% CI = 56.1–67.6%) of healthcare professionals worldwide had experienced any form of WPV. According to the Occupational Safety and Health Administration (OSHA), healthcare positions were among the most violence-prone industries in the USA, excluding law enforcement. Between 2011 and 2013, an average of 24 000 annual violent acted against employed adults occurred annually, with 75% of these incidents taking place in health-care settings [3]. In China, the incidence of WPV among medical staff ranged from 47.93 to 83.3%, with verbal violence and mental abuse being the most common forms [4–6]. Emergency department staff reported higher frequencies of WPV exposure than other practice settings [2]. Compared to other departments, the emergency department, positioned as the hospital’s frontline, managed the highest concentration of critically ill patients, a broad spectrum of diseases, and carried the heaviest workload in terms of urgent care and patient management. The limited time for effective communication with patients and their families further contributed to the heightened occurrence of WPV in the setting.

Due to prolonged exposure to high-pressure environments and elevated risks of WPV, health-care professionals in EDs were more likely to develop adverse psychological outcomes, among which burnout was one of the most common manifestations [7]. Maslach et al. defined occupational burnout as ‘a psychological syndrome resulting from prolonged emotional and interpersonal stressors associated with work’ and divided it into three dimensions: emotional exhaustion (EE), depersonalisation (DP), and reduced personal accomplishment (RPA) [8]. Previous studies also indicated that health-care professionals are at high risk for occupational burnout. Aiken et al. conducted a survey of nurses in 1406 hospitals across nine countries from 1999 to 2009, revealing that burnout was widespread among medical staff, with rates reaching as high as 60% in both Japan and South Korea [9]. Liu et al. undertook a comprehensive study on workplace violence, job satisfaction, occupational burnout, organisational support, and turnover intention among healthcare workers from nine tertiary hospitals in eastern, central, and western China [7]. The results indicated that 69.1% of the respondents experienced a high level of occupational burnout, and the levels of all three dimensions of burnout were significantly associated with the prevalence of workplace violence.

Under the dual stress of WPV and the resulting job burnout, healthcare workers may develop depressive symptoms. Rudkjoebing et al. conducted a meta-analysis to thoroughly assess the association between WPV and negative psychological outcomes, particularly depressive symptoms [10]. They found that the relative risk (RR) for depressive symptoms associated with WPV was 1.42 (95% CI = 1.31–1.54, *I*^2^ = 0%). Yang et al. investigated the situation of WPV against doctors in tertiary hospitals in Guangzhou and the doctor’s depressive symptoms [11]. They discovered that the doctors who experienced WPV had a significantly higher incidence of depressive tendencies compared to those who had not encountered violence.

According to a study on the prevalence of burnout and its influencing factors among health-care professionals in China, the prevalence of burnout among health-care professionals in five provinces was 58.0%, with 31.8% experiencing occupational stress and 31.0% showing depressive symptoms. After excluding the influence of various factors, stepwise linear regression analysis showed that depressive symptoms and occupational stress were both independent influencing factors of burnout, with depressive symptoms and the dimensions of social support and organisation and reward of occupational stress having significant influences on burnout. Depressive symptoms exhibited mediating effects on the relationship between occupational stress and burnout, with contribution proportions of the mediating effects being 41.00% and 59.45%, respectively [12]. Furthermore, a study conducted across 23 provinces in China has indicated that health-care professionals who have experienced WPV in the past year had doubled the risk of developing high levels of occupational burnout compared to those without such experiences [13].

Currently, studies on the correlation between WPV and symptoms of burnout and depressive symptom primarily employ statistical methods such as correlation and regression analyses [13–15]. These traditional models had a few limitations in handling complex theoretical frameworks. Specifically, when the research focus involved psychological constructs that cannot be directly measured, constructs typically formed as latent variables through multiple scale items, and the exploration of potential complex causal pathways among variables (such as mediating or moderating effects), structural equation modelling (SEM) demonstrated its unique advantages. Yet, the relationship among these factors was not clear, and studies were rarely based on a national representative sample [6,11,16]. The use of a national sample can effectively enhance the statistical representativeness of the sample for emergency department healthcare workers in China, thereby strengthening the generalisability and external validity of research findings. By covering research sites with diverse geographical regions, economic development levels, and medical resource allocation characteristics, this approach significantly reduced regional selection bias and reinforced the robustness and reliability of research conclusions. Therefore, this study aimed to build a SEM upon existing correlation analysis results on a large-scale national sample, explore the specific strengths and pathways of associations among WPV, burnout and depressive symptom. By elucidating the mechanisms linking WPV, burnout and depressive symptoms, the findings of this study will help to inform the development of effective intervention measures to reduce the incidence of burnout and depressive symptoms. This will not only enhance employees’ psychological health and job satisfaction, but also contribute to the overall health and sustainable development of the organisation.

First and foremost, a theoretical model needs to be established. Previous studies had indicated that burnout, as an emotional experience, may serve as a mediating role between WPV and depressive symptom. Specifically, a study by Jung et al. among Korean workers demonstrated that burnout partially mediated the relationship between job stress and depressive symptom [17]. Wu et al. provided a clear analytical framework by using SEM to examine the relationships among work environment, WPV, job burnout, intention to leave, and job satisfaction in nurses [18]. Results showed that WPV had a significant positive effect on high job burnout and negative psychological states (low professional identity, high intention to leave), with job burnout mediating this association, offering reference for our study, but the sample was limited to Guangdong nurses. In addition, WPV measurement only focused on verbal/physical abuse (excluding psychological violence), and negative outcomes were assessed via observed variables rather than latent constructs, potentially limiting characterisation of psychological constructs' multidimensionality. Therefore, the following hypothetical model is constructed, and the following hypotheses are proposed (**Figure 1**):

**Figure 1:**
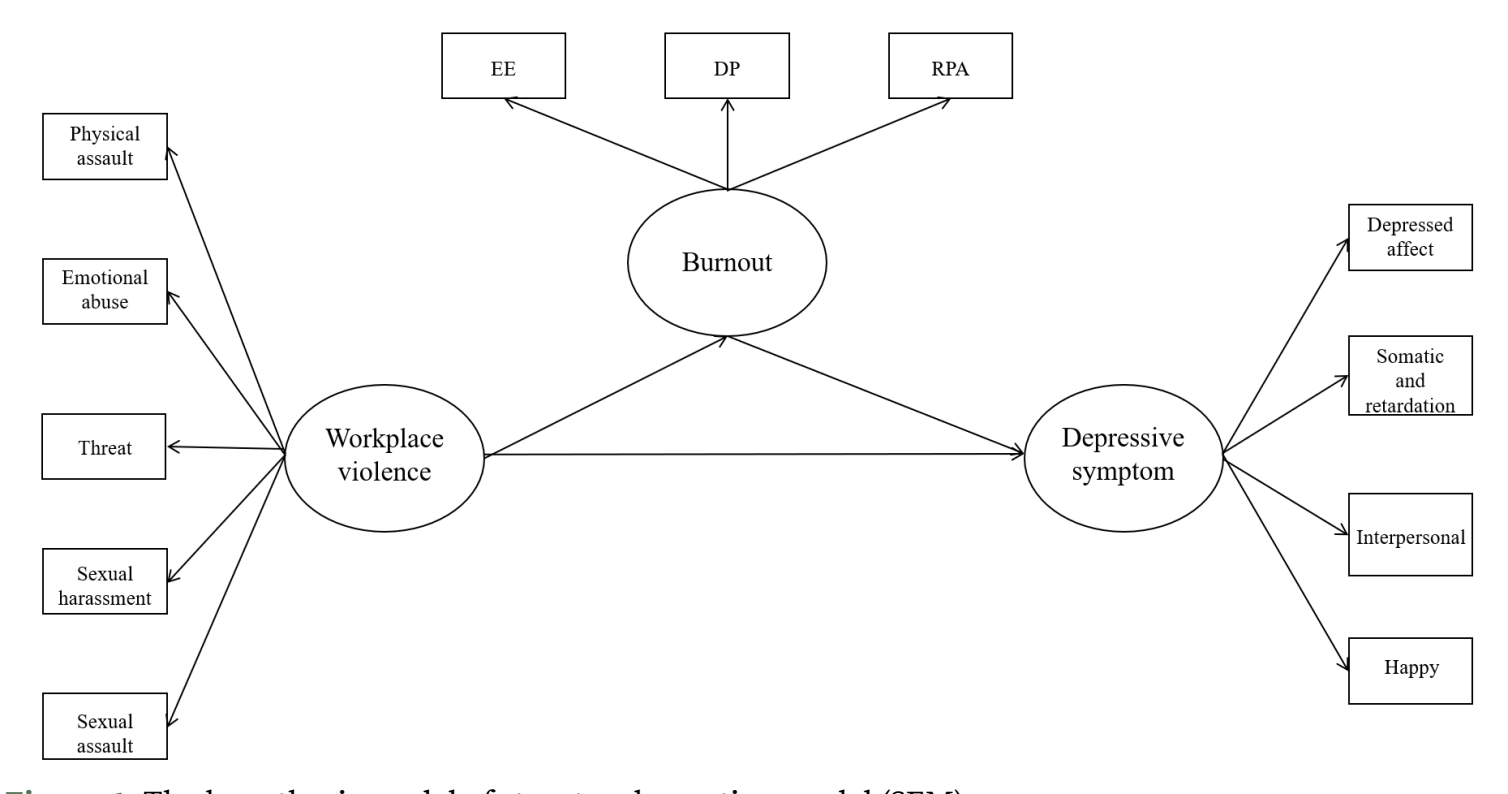
The hypothesis model of structural equation model (SEM).

Hypothesis 1 (H1): the level of WPV experienced by emergency department physicians is positively correlated with the level of depressive symptom.

Hypothesis 2 (H2): burnout mediates the relationship between WPV and depressive symptoms among emergency department physicians.

## METHODS

### Data sources

There are 22 provinces, five autonomous regions, and four municipalities of mainland China (not including Hong Kong, Macao, and Taiwan), and China is usually divided into eastern, central, and western regions. A multistage stratified random sampling design was used in this study. First, the 31 provinces, autonomous regions, and municipalities in China were categorised as high-developed, medium-developed, and low-developed regions according to per capita disposable income in 2018. Second, 10 hospitals were randomly selected from each province/autonomous region/municipality. Third, according to the number and scale of the hospitals, from each sampled hospital, 40% of the emergency physicians who had practiced in the EDs for at least six months were randomly selected to complete a self-administered questionnaire. A simple random sampling method was used at each stage. The eligibility criteria of participants were:

1) physicians practiced in EDs;

2) worked for at least six months.

In total, 15 455 emergency physicians were asked to participate in this survey, and 182 physicians did not respond. In addition, 30 questionnaires were discarded, because information on WPV was missing. Given the purpose of investigating the prevalence of WPV in the past year, we further excluded 395 emergency physicians whose work tenure was less than one year. Ultimately, 14 848 eligible questionnaires were used in this analysis, yielding a response rate of 93.18%. According to Kline [19], the sample size-to-parameter ratio in SEM should not be lower than 10:1. With 45 estimated parameters in the current model, the minimum required sample size was calculated as 450 cases. Considering a potential 10% non-response rate, the final sample size was 495.Thus, the sample size of this study was appropriate to test the model.

The study protocol was approved by the Institutional Ethics Board of the Second Affiliated Hospital of Hainan Medical University, Haikou, China (HYLL-2018-035). All individuals provided written informed consent to the researchers before the survey, and participants’ personal information was kept confidential.

### Measurement of WPV

WPV was measured via a scale developed by Wang et al. [20], which assessed whether participants experienced any form of WPV and related situations in the past 12 months. This scale had shown good reliability and validity when used with health-care professionals [21,22]. This survey included five items that inquire about participants' experiences in the past year regarding physical assault, emotional abuse, threat, sexual harassment, and sexual assault in the workplace, with each item followed by a brief description to aid participants' comprehension. The responses were rated using a 4-point Likert scale: ‘0 = none, 1 = once, 2 = two to three times, 3 = more than three times.’ An affirmative response to any item (other than ‘0’) indicated the presence of WPV. In this study, the questionnaire had a Cronbach’s α of 0.80 and a KMO value of 0.75, indicating satisfactory reliability.

### Measurement of burnout

This study utilised the Maslach Burnout Inventory-Human Services Survey (MBI-HSS), which was localised and revised by Li and Shi [23] to assess occupational burnout among emergency department physicians. This authoritative tool for assessing burnout consists of 22 items divided into three dimensions: Emotional Exhaustion (EE) with nine items, Depersonalisation (DP) with five items, and Reduced Personal Accomplishment (RPA) with 8 items. Responses were evaluated using a 7-point Likert scale. In accordance with the diagnostic criteria for burnout norms among Chinese nursing populations [24], cut-off scores of 27 for EE, 8 for DP, and 24 for RPA were used to delineate the severity of burnout, with all three dimensions negative indicating no burnout; one dimension positive indicating mild; two dimensions positive indicating moderate; and all three dimensions positive indicating severe [25]. In this study, the Cronbach’s α coefficients for the MBI total scale and the EE, DP, and RPA subscales were 0.90, 0.96, 0.85, and 0.91, respectively; the KMO values were 0.94, 0.94, 0.86, and 0.91, respectively. These above results indicated that the questionnaire had a good reliability.

### Measurement of depressive symptom

The measurement of depressive symptoms among emergency department physicians utilised the Center for Epidemiologic Studies Depression Scale (CES-D), which primarily assessed the frequency of depressive symptoms over the past week. The scale consisted of 20 items across four dimensions: Depressed Affect with eight items, Happy with four items, Somatic and Retardation with 6 items, and Interpersonal with two items. The scale had been evaluated for reliability and validity and normed for different age groups in Chinese urban population [26]. Responses to each question include: ‘Rarely or none of the time (less than 1 day)’; ‘Some or a little of the time (1–2 days)’; ‘Occasionally or a moderate amount of time (3–4 days)’; ‘Most or all of the time (5–7 days)’, with each frequency assigned a value from 0 to 3. The total score ranges from 0 to 60, with higher scores indicating a higher frequency of depressive symptoms, and a score >28 was considered indicative of a depressive state [27]. In this study, the Cronbach’s α coefficients for the CES-D total scale and the four subscales were 0.97, 0.93, 0.89, 0.88, and 0.87, respectively, and the KMO values were 0.98, 0.94, 0.84, 0.89, and 0.5, respectively. The Interpersonal dimension had a lower KMO value, but since it only contained two items, the overall reliability of the questionnaire was still considered good.

### Data analysis

Data analyses including SEM, were conducted using STATA 17.0 (StataCorp LLC, College Station, TX, USA) and Amos 26.0 (IBM Corp, Armonk, NY, USA) software. Pearson correlation coefficients were calculated to test the associations between variables. Analysis of variance was used to verify whether there were differences in levels of depressive symptoms among populations with different characteristics, with a significance level of α = 0.05. The fit indices for the SEM included the Goodness of Fit Index (GFI), Adjusted Goodness of Fit Index (AGFI), Normed Fit Index (NFI), Incremental Fit Index (IFI), Comparative Fit Index (CFI), Parsimonious Normed Fit Index (PNFI), Parsimonious Comparative Fit Index (PCFI), and Root-mean-square Error of Approximation (RMSEA). A model is considered to have a good fit if GFI, AGFI, NFI, IFI, and CFI values are greater than 0.90, PNFI and PCFI values are greater than 0.05, and RMSEA values are less than 0.06.

## RESULTS

### Characteristics

In this study, a total of 14 848 valid survey data from emergency department physicians were included. Majority of the respondents were male (70.53%), with about half (50.88%) of them aged between 35 to 49 years. Regarding workplace affiliation, the vast majority of physicians (95.80%) were employed by public hospitals, with the highest proportion of doctors from tertiary hospitals, accounting for 66.81%. There were statistically significant differences in depressive symptom levels among populations of different ages, hospital types, hospital levels, shift work status, work stress levels, and years of experience in emergency medicine (all *P* < 0.05) (**Table 1**).

**Table 1:** Basic characteristics of the survey participants

Variable	Number (n)	Percentage (%)	Depressive symptom scores (mean ± SD)	*P-*value*
**Gender**				0.26
Male	10 472	70.53	16.84 ± 14.87	
Female	4376	29.47	16.71 ± 14.57	
**Age (years)**				<0.01
18–34	5775	38.89	17.51 ± 14.81	
35–49	7555	50.88	17.15 ± 14.82	
50–64	1509	10.16	12.32 ± 13.73	
≥65	9	0.07	15.00 ± 15.55	
**Hospital type**				<0.01
Public hospitals	14 224	95.80	16.92 ± 14.85	
Private hospitals	624	4.20	14.01 ± 12.81	
**Hospital hierarchy**				<0.01
Tertiary hospitals	9920	66.81	17.02 ± 14.73	
Secondary hospitals	4691	31.59	16.55 ± 14.94	
Others	237	1.60	12.50 ± 13.23	
**Shift work**				<0.01
Yes	12 956	87.26	17.66 ± 14.95	
No	1892	12.74	10.93 ± 12.07	
**Work pressure**				<0.01
None	63	0.42	5.09 ± 10.63	
Low	385	2.59	6.03 ± 8.28	
Middle	2023	13.62	7.33 ± 9.26	
Middle-high	7964	53.64	14.56 ± 11.96	
High	4413	29.72	26.29 ± 16.68	
**Work tenure (years)**				<0.01
<10	9462	63.73	17.12 ± 14.72	
10~	5275	35.53	16.38 ± 14.90	
≥30	111	0.75	9.60 ± 12.66	

SD – standard deviation, WPV – workplace violence

*P denotes mean differences in depressive symptom scores among different subcategories.

### Descriptive and correlational analysis of study variables

A total of 90.40% of emergency physicians reported experiencing at least one instance of WPV in the past year, with emotional abuse being the most common form (87.25%), followed by threats (71.09%), physical violence (48.24%), verbal sexual harassment (38.13%), and physical sexual harassment (19.37%). The mean burnout score for emergency physicians was 82.94 ± 26.11, with 94.41% of emergency physicians exhibiting signs of burnout. This included mild burnout in 21.52%, moderate burnout in 24.33%, and severe burnout in 48.55%. The detection rates for emotional exhaustion, depersonalisation, and low personal accomplishment were 61.47, 64.49, and 89.90%, respectively. Additionally, 19.63% of emergency physicians presented symptoms of depression.

The correlational analysis between WPV, burnout, and depressive symptoms is presented in **Table 2**, showing that the correlations between each pair of the three study variables were statistically significant (*P* < 0.01).

**Table 2:** Correlational analysis of study variables

Variable	Mean ± SD	Range	WPV	Depression	Burnout
WPV	5.65 ± 4.06	0–15			
Depressive symptom	16.80 ± 14.78	0–60	0.444*		
Burnout	82.94 ± 26.11	0–154	0.347*	0.562*	

WPV – workplace violence

*P<0.01.

In this study, job burnout status and depressive symptom levels were considered as dependent variables. Variables with statistically significant results (*P* < 0.05) from the univariate analysis (**Table 1**) along with WPV exposure status, were incorporated as independent variables to multiple linear regression. Notably, work tenure was moderately positively correlated with age (Pearson correlation coefficient = 0.59, *P* < 0.01). To avoid the impact of multicollinearity on model stability, work tenure was not included in this multivariable model.

To precisely elucidate the impact of different types of violence on job burnout and depressive symptoms, the sample was restricted to participants without exposure to WPV and exposure to only one type of WPV, resulting in a final analytical sample of 3542 participants. Results showed that work pressure reported a significant predictive effect on both job burnout (*B* = 0.36) and depressive symptoms (*B* = 4.61), respectively. Among different types of violence, emergency department physicians who experienced emotional abuse had significantly higher odds of job burnout (*B* = 0.45) and depressive symptoms (*B* = 2.99) compared with unexposed counterparts. Except for emotional abuse, no significant associations were found between other types of violence and job burnout. Additionally, sexual harassment (*B* = 2.24) significantly increased the odds of depressive symptoms (**Table 3**).

**Table 3:** Multiple linear regression of predictors of job burnout and depressive symptom among Chinese emergency department physician

Variable	Burnout			Depressive symptom		
	***B***	***t***	***P-*value**	***B***	***t***	***P-*value**
**Age**	−0.13	−5.12	﹤0.001	−1.60	−6.01	﹤0.001
**Work pressure**	0.36	18.41	﹤0.001	4.61	22.54	﹤0.001
**Hospital type (ref: public hospitals)**						
Private hospitals	−0.15	−2.06	0.04	−0.42	−0.53	0.60
Hospital hierarchy (ref: tertiary hospitals)						
Secondary hospitals	−0.14	−4.20	﹤0.001	−0.56	−1.55	0.12
Others	−0.20	−1.99	0.05	0.52	0.50	0.62
**Shift work (ref: yes)**						
No	−0.04	−1.07	0.30	−0.66	−1.45	0.15
**WPV type (ref: never)**						
Physical assault	−0.04	−0.33	0.75	2.26	1.63	0.10
Emotional abuse	0.45	13.71	﹤0.001	2.99	8.55	﹤0.001
Threat	0.04	0.50	0.61	0.25	0.31	0.76
Sexual harassment	0.18	1.68	0.09	2.25	1.96	0.05
Sexual assault	0.11	0.27	0.80	4.29	0.99	0.32

WPV – workplace violence

### SEM analysis result

**Figure 2:**
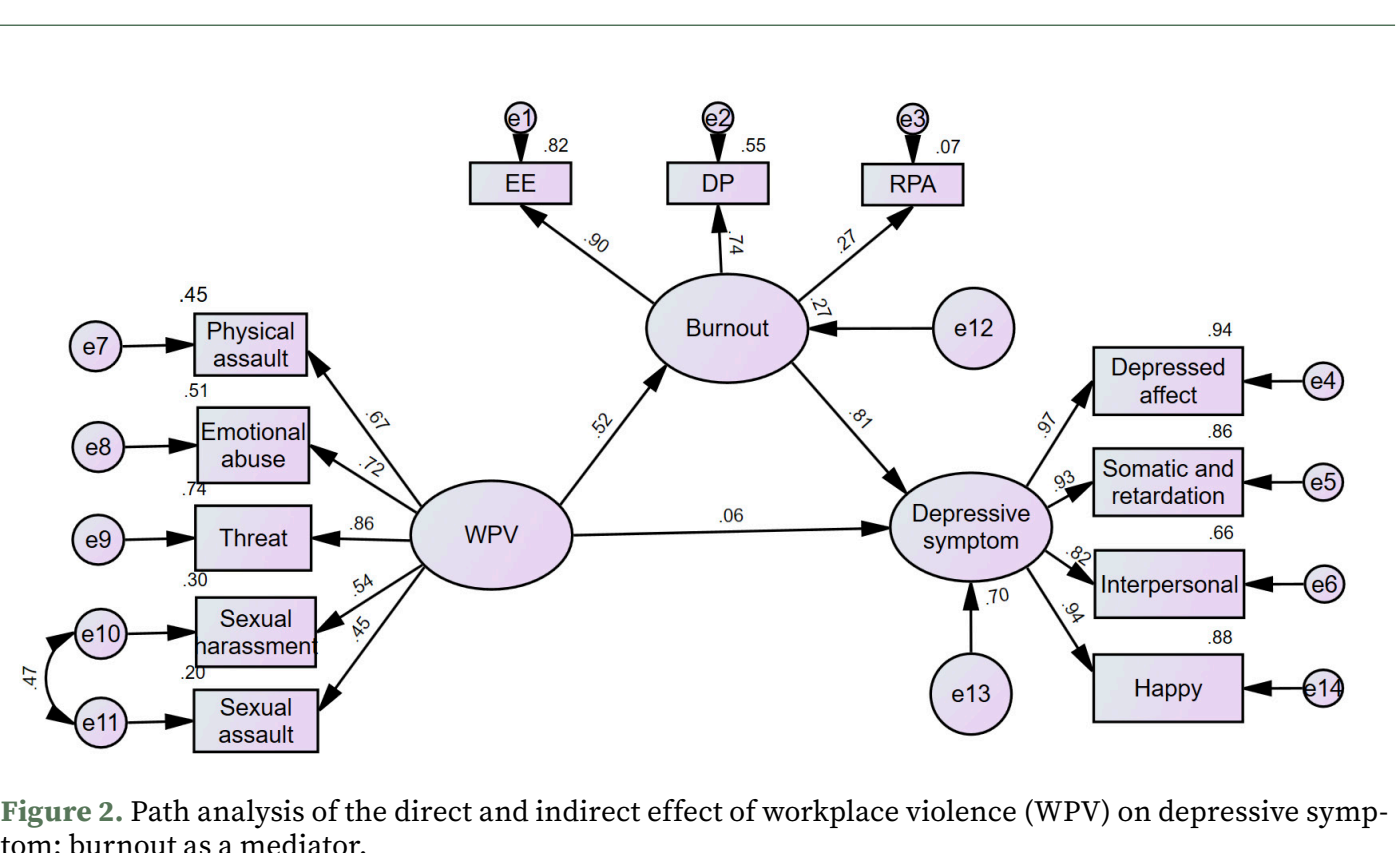
Path analysis of the direct and indirect effect of workplace violence (WPV) on depressive symptom: burnout as a mediator.

The SEM hypothesis was constructed using WPV, burnout, and depressive symptoms as latent variables. The observed variables corresponding to each dimension included the frequency of the five forms of violence on the violence scale (physical assault, emotional abuse, threats of intimidation, verbal harassment, and physical harassment), the three dimensions of the MBI-HSS (emotional exhaustion, depersonalisation, and low personal accomplishment), and the four dimensions of the CES-D (emotional disturbance, somatic symptoms, positive emotions, and interpersonal conflict). The maximum likelihood method was used to verify the hypothesis, and after minor adjustments, the final standardised path diagram and coefficients is shown in **Figure 2**.

The model fit results indicated that the absolute fit indices are within the ideal criteria, with GFI = 0.93 and RMSEA = 0.09, and AGFI = 0.896, which was close to the standard of 0.90. In terms of incremental fit indices, NFI, IFI, and CFI are 0.956, 0.957, and 0.957, respectively, all of which exceed the standard value of 0.90, indicating an ideal state. For the parsimony fit indices, PNFI and PCFI are both 0.725, which was above the standard value of 0.05, suggesting a good model fit. Overall, the experimental research model in this study, with minor modifications, showed a high degree of credibility in the fit results obtained.

The results of the effect analysis of the SEM revealed that the factor loadings for the five types of violence experienced by emergency department physicians were 0.67, 0.72, 0.86, 0.54, and 0.45, respectively. Among these, the factor loading for threats and intimidation was the highest, indicating that exposure to threats and intimidation has the greatest impact on the perception of violence among emergency department physicians. For each one standard deviation increase in threats and intimidation, the perception of violence increased by an average of 0.86 standard deviations.

In the context of burnout among emergency department physicians, the factor loadings for the three dimensions of emotional exhaustion, depersonalisation, and reduced personal accomplishment were 0.90, 0.74, and 0.27, respectively. The highest factor loading was observed for emotional exhaustion, suggesting that it had the most significant influence on burnout among emergency department physicians. For each one standard deviation increase in emotional exhaustion scores, the level of burnout increased by an average of 0.90 standard deviations.

Regarding depressive symptom, the factor loadings for the four dimensions of depression, somatic symptoms, interpersonal conflict, and happy were 0.97, 0.93, 0.82, and 0.94, respectively. The highest factor loading was for depression, with each one standard deviation increase in this dimension leading to an average increase of 0.97 standard deviations in the severity of depressive symptoms among emergency department physicians.

Overall, among emergency department physicians, WPV had a significant positive impact on burnout (*P* < 0.01), with each one standard deviation increase in WPV leading to an average increase of 0.52 standard deviations in burnout levels, thus validating H1. Burnout significantly and positively affects the level of depressive symptoms (*P* < 0.01), with each one standard deviation increase in burnout levels leading to an average increase of 0.81 standard deviations in depressive symptom levels.

Additionally, the direct effect of WPV on depressive symptoms was statistically significant (*P* < 0.01), confirming H2; however, the standardised coefficient between the two was only 0.06, indicating a relatively weak direct relationship between WPV and depressive symptoms.

## DISCUSSION

The findings of this study demonstrate that the frequent occurrence of WPV among emergency department physicians indirectly increases the risk of depressive symptoms by exacerbating burnout. Through the analysis of SEM, this study validated the mediating effect model of job burnout in the association between WPV and depressive symptoms, providing empirical evidence for the applicability of this theoretical framework among the Chinese emergency department physician population.

WPV is considered a multidimensional concept, encompassing various forms such as physical assault, verbal abuse, threats, and harassment. In this study, the incidence rate of WPV among emergency department physicians reached 90.40%, which is significantly higher than the average global incidence rate of WPV among health-care professionals reported by Liu et al. [2] in a previous meta-analysis (62.3%). The reasons for this situation are manifold. Firstly, in the high-pressure environment of the emergency department, physicians frequently confront life-and-death decisions and the emotional fluctuations of patients' families, rendering them high-risk targets for WPV [28]. Secondly, the scarcity of emergency medical resources is also a significant factor. Xu et al. conducted a questionnaire and telephone survey across all non-military tertiary general hospital emergency departments in Beijing, revealing that the development of emergency departments in Beijing's tertiary general hospitals is highly imbalanced, with a turnover rate of emergency physicians reaching 62.7% within three years [29]. In this study, only 36.27% of physicians had been working in the emergency department for over a decade. Thirdly, the lack of beds and personnel leads to prolonged waiting times for patients in the emergency department, a condition that not only may exacerbate patients' dissatisfaction but also increases their mortality rate [30]. Against this backdrop, the risk of emergency physicians experiencing WPV correspondingly increases [31].

Experiences of WPV can lead to persistent work-related stress and emotional exhaustion, which, if unmanaged, may evolve into burnout. In this study, up to 94.41% of respondents exhibited varying degrees of burnout, a figure higher than that reported in relevant Chinese studies post-COVID-19 pandemic [13,32], as well as the rates observed among emergency department nurses in the United States [33] and the Egypt [34]. SEM results indicated that for every one standard deviation increase in violent incidents, burnout levels increased by an average of 0.52 standard deviations, a finding consistent with results of previous research [18]. The results of MLR showed that compared with physicians not exposed to WPV, respondents who suffered from emotional abuse had a significantly higher odds of job burnout (*B* = 0.45). For doctors who experienced other types of WPV, there was no statistically significant difference in the odds of job burnout compared with the unexposed group. Within the dimensions of job burnout, the factor loading for emotional exhaustion (0.90) was significantly higher than the other two factors (0.74 and 0.27), indicating that emotional exhaustion contributes the most to burnout. These findings indicated that emotional abuse exerts a significant positive predictive effect on job burnout, suggesting that prevention efforts for job burnout should prioritise emergency department physicians who had experienced emotional abuse. For those who had developed job burnout, intervention strategies should focus on precise intervention for the emotional exhaustion dimension, such as implementing Mindfulness-Based Cognitive Behaviour Therapy (MBCT)-based intervention programmes.

Burnout mediates the relationship between WPV and depressive symptoms, potentially influencing an individual's psychological resources and coping strategies, which in turn affect their levels of depressive symptoms. In this study, 19.63% of respondents exhibited depressive symptoms, which is lower than the 25.2% reported by Song et al. [35] in their 2020 survey in Hubei, China. This discrepancy may be related to the timing of the surveys, as our study collected data from July to September 2019, just before the outbreak of the COVID-19 pandemic. Since the pandemic's onset, frontline health-care workers had faced a sharp increase in work pressure, which may exacerbate their mental health issues. The study also found that the probability of exhibiting depressive symptoms decreases with age, a result consistent with the findings of Song et al. [35] and Roberts et al. [36]. The underlying mechanisms need further exploration. Specifically, physicians in their prime years bear the heaviest workload in the emergency department [37]. With age, physicians experience a gradual reduction in work demands (such as night shift frequency), while occupational resources continue to accumulate. Senior physicians demonstrated more robust social support networks, richer occupational resource reserves, and higher self-efficacy, enabling them to exhibit stronger resilience in coping with work-related resource loss events [38]. Another plausible explanation was the ‘healthy worker effect’, where emergency department physicians with low psychological resilience might exit the profession early in their careers, leading to a predominance of high-resilience individuals among survivors. This process created a statistical association between longer work tenure and lower levels of job burnout and depressive symptom [39].

The SEM results indicated that WPV is a positive predictor of depressive symptoms. Although the direct effect was statistically significant, the standardised path coefficient between the two was only 0.05. In contrast, the indirect effect mediated by burnout was more pronounced. This finding revealed the lag effect and transformation mechanism of WPV hazards, indicating that its impact on mental health was not derived from direct violent shock but realised through the induction of long-term chronic job burnout. The results provided empirical evidence for the Conservation of Resources (COR) theory [40] and the Job Demands-Resources (JD-R) model [41]. The core implication is that any intervention programme aimed at mitigating the impact of WPV on depressive symptom must prioritise the prevention and alleviation of job burnout as its central goal. Merely addressing WPV incidents themselves may fail to interrupt the key pathway leading to depression. Therefore, for healthcare workers who have experienced WPV, a systematic and continuous dual-dimensional monitoring mechanism for burnout and depressive symptom should be established. Following a WPV incident, in addition to routine incident management, it is necessary to simultaneously conduct burnout risk assessment and psychological intervention, constructing a three-tiered progressive support system: immediate crisis intervention → medium-term burnout prevention and control → long-term safe work environment creation.

The strengths of this study lay in its nationwide sample across China, encompassing multiple levels and types of hospitals, with a wide age range of respondents, indicating good sample representativeness. However, the study had certain limitations. First, the survey method used was an online self-administered questionnaire, which may introduce information bias. Second, since this study selected emergency department physicians, the results may not be generalisable to all health-care personnel. Of note, the cross-sectional design precluded tracking the dynamic changes in depressive symptoms and job burnout before and after WPV exposure, severely limiting the temporal inference of causal relationships. More importantly, job burnout and depressive symptoms exhibited substantial overlap in conceptual dimensions and temporal sequences, with complex bidirectional associations. The results cannot rule out the possibility of reverse causality, whereby current depressive symptom levels may positively influence individuals' perception of job burnout and even interfere with the accuracy of recall and subjective severity assessment of WPV experiences. This potential confounding effect suggests that burnout might act as a confounder rather than a true mediator in the relationship between depressive symptoms and WPV. Therefore, the mediational analysis results based on cross-sectional data should be interpreted cautiously.

## CONCLUSIONS

This study provides evidence for the relationship between WPV, burnout, and depressive symptoms among emergency department physicians. It is recommended that managers establish a full-chain intervention mechanism. Firstly, implementing source control of WPV exposure, with a focus on curbing emotional abuse and improving workplace safety assurance systems. Secondly, adopting a hierarchical job burnout intervention strategy, including dynamic workload management and psychological resilience enhancement programmes for young physicians, and establishing a professional psychological mentoring system and group mindfulness therapy training. Thirdly, addressing the long-term resource depletion caused by WPV by constructing a systematic and continuous dual-dimensional monitoring mechanism for burnout and depression.
